# Concomitant Socioeconomic, Behavioral, and Biological Factors Associated with the Disproportionate HIV Infection Burden among Black Men Who Have Sex with Men in 6 U.S. Cities

**DOI:** 10.1371/journal.pone.0087298

**Published:** 2014-01-31

**Authors:** Kenneth H. Mayer, Lei Wang, Beryl Koblin, Sharon Mannheimer, Manya Magnus, Carlos del Rio, Susan Buchbinder, Leo Wilton, Vanessa Cummings, Christopher C. Watson, Estelle Piwowar-Manning, Charlotte Gaydos, Susan H. Eshleman, William Clarke, Ting-Yuan Liu, Cherry Mao, Samuel Griffith, Darrell Wheeler, for the HPTN061 Protocol Team

**Affiliations:** 1 The Fenway Institute of Fenway Health and the Infectious Disease Division of Beth Israel Deaconess Medical Center and Harvard Medical School, Boston, Massachusetts, United States of America; 2 Vaccine and infectious Disease Division, Fred Hutchinson Cancer Research Center, Seattle, Washington, United States of America; 3 Laboratory of Infectious Disease Prevention, New York Blood Center, New York, New York, United States of America; 4 Department of Medicine, Harlem Hospital and Columbia University, New York, New York, United States of America; 5 The Department of Epidemiology and Biostatistics, George Washington University, Washington, DC, United States of America; 6 Department of Global Health and Emory Center for AIDS Research, Emory University Rollins School of Public Health, Atlanta, Georgia, United States of America; 7 HIV Research Section San Francisco Department of Health, San Francisco, California, United States of America; 8 Department of Human Development, Binghamton University, Binghamton, New York, United States of America; 9 Department of Pathology, Johns Hopkins University School of Medicine, Baltimore, Maryland, United States of America; 10 Department of Medicine, Johns Hopkins University School of Medicine, Baltimore, Maryland, United States of America; 11 FHI 360, Durham, North Carolina, United States of America; 12 School of Social Work, Loyola University Chicago, Chicago, Illinois, United States of America; Chinese Academy of Sciences, Wuhan Institute of Virology, China

## Abstract

**Background:**

American Black men who have sex with men (MSM) are disproportionately affected by HIV, but the factors associated with this concentrated epidemic are not fully understood.

**Methods:**

Black MSM were enrolled in 6 US cities to evaluate a multi-component prevention intervention, with the current analysis focusing on the correlates of being newly diagnosed with HIV compared to being HIV-uninfected or previously diagnosed with HIV.

**Results:**

HPTN 061 enrolled 1553 Black MSM whose median age was 40; 30% self-identified exclusively as gay or homosexual, 29% exclusively as bisexual, and 3% as transgender. About 1/6^th^ (16.2%) were previously diagnosed with HIV (PD); of 1263 participants without a prior HIV diagnosis 7.6% were newly diagnosed (ND). Compared to PD, ND Black MSM were younger (p<0.001); less likely to be living with a primary partner (p<0.001); more likely to be diagnosed with syphilis (p<0.001), rectal gonorrhea (p = 0.011) or chlamydia (p = 0.020). Compared to HIV-uninfected Black MSM, ND were more likely to report unprotected receptive anal intercourse (URAI) with a male partner in the last 6 months (p<0.001); and to be diagnosed with syphilis (p<0.001), rectal gonorrhea (p = 0.004), and urethral (p = 0.025) or rectal chlamydia (p<0.001). They were less likely to report female (p = 0.002) or transgender partners (p = 0.018). Multivariate logistic regression analyses found that ND Black MSM were significantly more likely than HIV-uninfected peers to be unemployed; have STIs, and engage in URAI. Almost half the men in each group were poor, had depressive symptoms, and expressed internalized homophobia.

**Conclusions:**

ND HIV-infected Black MSM were more likely to be unemployed, have bacterial STIs and engage in URAI than other Black MSM. Culturally-tailored programs that address economic disenfranchisement, increase engagement in care, screen for STIs, in conjunction with safer sex prevention interventions, may help to decrease further transmission in this heavily affected community.

## Introduction

In the United States (US), sex between men is the most common mode of HIV transmission, and, Black men who have sex with men (MSM) have the highest HIV prevalence and incidence, compared to any other US subpopulation [1]–[2]. In 2009, MSM constituted 61% of new HIV infections diagnosed in the US, and HIV incidence increased by 48% among Black MSM aged 13 to 29 years old between 2006 and 2009 [1]. Prior HIV prevention research has suggested that increased self-reported risk behaviors do not solely account for the higher prevalence of HIV in Black MSM compared to other MSM [3]–[6]. For prevention interventions to be successful, public health officials and clinicians need to better understand specific contextual factors that are contributing to this concentrated epidemic.

Previous studies have often evaluated how specific socio-behavioral factors (such as partner mixing patterns, use of drugs during sex), structural factors (such as poverty, homelessness), or biological factors (such as concomitant sexually transmitted infections) each contribute to the disproportionate rates of HIV among Black MSM. The current analysis was designed to evaluate the interactions of these different factors, by comparing their prevalence among participants who were newly diagnosed with HIV, to those were previously diagnosed with HIV, and those who were HIV-uninfected at enrollment, in order to better understand contemporary potentiators of the disproportionate spread of HIV among Black MSM.

## Methods

HPTN 061 was a study of the HIV Prevention Trials Network (HPTN 061, also known as ‘The Brothers Study’), designed to assess the feasibility and acceptability of a multi-component intervention to reduce HIV incidence among Black MSM. The study recruited a sample of Black MSM in 6 US cities who were offered a program of HIV testing and sexually transmitted infection (STI) screening at enrollment and at 6 and 12 months, as well as referral for treatment of any new diagnosed infections. Participants were also offered peer health system navigation, in which a trained peer navigator assisted participants in accessing services for unmet medical and psychosocial needs identified at their enrollment visit. HPTN 061 was conducted in Atlanta, Boston, Los Angeles, San Francisco, Washington DC, and New York City (NYC). The institutional review boards at all participating institutions approved the study. This list of IRBs is also included as an appendix (File S1). Between July 2009 and October 2010, 1553 Black MSM were recruited directly from the community or as sexual network partners referred by index participants. Index participants were: (1) HIV infected, but unaware of their infection, or (2) previously diagnosed with HIV infection but not receiving HIV care and having unprotected sex with partners of negative or unknown HIV status, or (3) HIV-uninfected. Community recruitment methods were developed at each site and included venue-based outreach, engagement of key informants and local community-based organizations, print advertising, and use of online strategies including the placement of banner ads, text ads, chat room outreach, and social networking sites.

The goal for each city was to enroll 250 Black MSM recruited directly from the community who agreed to HIV testing. In order to obtain a sample of HIV-infected and uninfected Black MSM who were at increased risk for HIV acquisition or transmission, whether they were aware of their HIV status or not, enrollment of participants who were HIV-uninfected was capped at 200 participants at each site, and no more than 83 participants per site who refused HIV testing could be enrolled. An enrollment cap of 10 was applied to community-recruited participants with a prior diagnosis of HIV infection who were already in care, or reported only having unprotected anal sex with HIV-positive partners, since they would be at decreased risk for HIV transmission.

Men were eligible to participate in the study if they: self-identified as a man or were male at birth, and self-identified as Black, African American, Caribbean Black, or multiethnic Black, were at least 18 years old, reported at least one instance of unprotected anal intercourse with a man in the past six months, resided in the metropolitan area where the study was being conducted, did not plan to move away during the time of study participation, and provided informed consent for the study. Men were ineligible if they were enrolled in any other HIV interventional research study, if they had participated in an HIV vaccine trial or were a community-recruited participant in a category that had already reached its enrollment cap. Prescreening to determine eligibility was performed either in person or over the telephone.

At the enrollment visit, eligibility was confirmed and written informed consent obtained. Participants provided locator information as well as demographic information to an interviewer and then completed a behavioral assessment using audio computer-assisted self-interview (ACASI) technology. Following completion of the ACASI assessment, a social and sexual network questionnaire was completed with an interviewer.

### HIV and STI Testing

A rapid HIV antibody test was conducted after participants received HIV/STI risk-reduction counseling. If the rapid HIV test was reactive, HIV infection was confirmed at study sites by Western blot testing. Participants with HIV infection had CD4 cell count testing and HIV viral load testing performed. Quality assurance testing was performed retrospectively at the HPTN Network Laboratory to confirm the HIV infection status of all study participants at enrollment and to confirm cases of HIV seroconversion. For participants with low or undetectable HIV RNA who did not report a prior HIV diagnosis, enrollment samples were tested for the presence of antiretroviral drugs after the end of the study; men whose samples contained antiretroviral drugs indicative of antiretroviral therapy were considered to have a prior HIV diagnosis. Urine and rectal swabs were collected for *Neisseria gonorrhea*e (GC) and *Chlamydia trachomatis* (CT) testing (Hologic Gen-Probe Aptima Combo 2, San Diego, CA), and a blood specimen was collected for syphilis testing. All participants who had a positive test for any infection were referred for treatment, and medical and social services.

### Interviewer Administered Questions

Demographic characteristics were collected by an interviewer and included standard measures for age, sexual identity, education, employment, income, and student status.

### ACASI Administered Questions

The study used ACASI to collect data on HIV testing history, testing location, and reasons for testing. The ACASI interview also collected data on sexual risk behaviors in the 6 months prior to enrollment, including number of male, female and transgender partners, number new partners, HIV status and race/ethnicity of partners, partner type, number of receptive and insertive anal sex acts, number of sex acts that were protected by condoms and exchange of sex for money, drugs, or goods. Measures on alcohol use frequency, amount and dependency were derived from the Alcohol Use Disorders Identification Test [7]. The answers to 10 questions were scored on a point system (from 0 to 40) and a score of more than eight was used to indicate an alcohol problem. Questions on other substance use in the 6 months prior to enrollment included use of marijuana; inhaled nitrates; smoked and powder cocaine; methamphetamine; heroin; non-prescribed opiates; sedatives; hallucinogens; and injection drug use. For each drug, follow-up questions were included on number of days of use in the prior 30 days, and use in conjunction with protected and unprotected anal intercourse.

The Center for Epidemiologic Studies Depression Scale (CES-D), a 20-item, 7-point Likert type scale, was used to measure symptoms of depression [8]. The sum of all the scores was computed for participants who answered at least 19 items. A participant with a score of 16 or higher was considered as having depressive symptoms. Internalized homophobia was measured with a 7-item scale adapted from Herek and Glunt [9]. Responses were collected in a 5-point scale ranging from “disagree strongly” to “agree strongly” (α = 0.91). The sum of the seven scores was computed for each participant with complete answers to all seven questions. Participants were categorized as having low (score ≤16) or medium (score from 17–26) or high internalized homophobia (sum >26). Questions were asked about religious affiliation when growing up and current affiliation status.

### Statistical Analysis

Baseline socio-demographic, psychosocial, behavioral, and clinical characteristics were summarized by participants’ enrollment HIV status: HIV-uninfected, newly diagnosed, or previously diagnosed. Participants who were newly diagnosed were compared with those who were previously diagnosed and those who were HIV-uninfected using Chi-Square tests or Fisher’s Exact test for categorical variables, and Wilcoxon Rank-Sum test for continuous variables. Separate multivariate logistic regression models were used to assess associations between baseline characteristics and being newly diagnosed with HIV, versus being previously diagnosed with HIV and HIV-uninfected participants, respectively. Only covariates that were found to have a p-value of <0.10 in bivariate analysis were included in the multivariate models. SAS® version 9.2 statistical software was used to perform all analyses.

## Results

### Study Population

**Figure 1:**
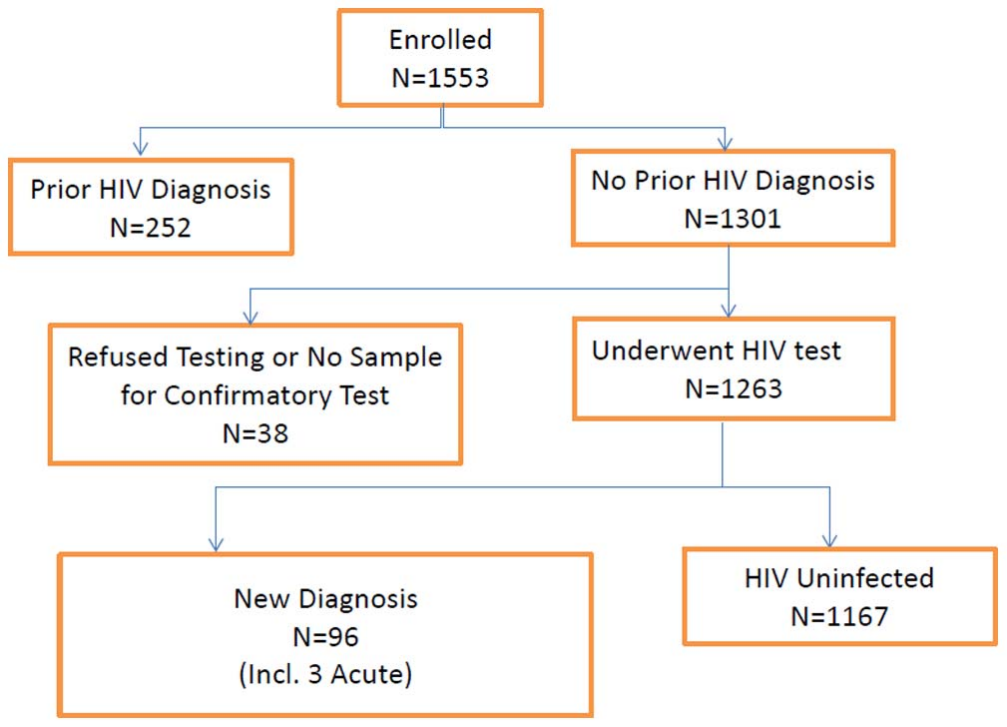
HIV serostatus of Black Men who have sex with men who enrolled in HPTN 061.

Of the 1,553 Black MSM who enrolled in HPTN 061, 252 (16.2%) either reported a prior HIV diagnosis or were considered to be previously diagnosed based on detection of antiretroviral drugs in their enrollment samples (see Methods). Among the 1,301 remaining men, 38 refused testing or had no sample available to confirm their HIV infection status. The remaining 1,263 men included 96 men (7.6%) who were newly diagnosed with HIV infection (including three who had acute HIV infection at enrollment) and 1,167 (92.4%) men who were HIV-uninfected (Figure 1).

The median age of all enrollees for whom HIV status was determined was 40 years, with 33% being between 18 and 30 years old (Table 1). Eight percent also identified as Latino, and 96% were US born; 55% had less than a college education, while 20% were currently students. Thirty-one percent were currently working, 10% lacked stable housing, and 38% of the participants had an annual household income of less than $10,000 a year, with around half having an income between $10,000 and $49,999 (Table 1). About one third of the participants reported that they had insufficient income once in a while, and 24% reported that their income was insufficient fairly or very often. Only 11% of the men were married or reported having a primary partner. Twenty percent of the men were enrolled in NYC, 19% in Atlanta, 19% in Los Angeles, 14% in Washington, DC, 15% in Boston, and 13% in San Francisco. Compared to men who were previously diagnosed with HIV, men who were newly diagnosed with HIV were significantly more likely to be younger (median age 37 vs. 43, *p*<0.001) (Table 1). Compared to men who were HIV-uninfected at enrollment, men who were newly diagnosed with HIV were less likely to be working (21% vs. 35%, *p* = 0.011), less likely to have college education (35% vs. 46%, p = 0.049), and were less likely to report not having enough money for rent, food, or utilities (14% vs. 25%, p = 0.030).

**Table 1:** Sociodemographic characteristics of HPTN 061study participants by HIV status: Newly diagnosed versus previously diagnosed or HIV-uninfected.

Variable	Total	Newly Diagnosed(N = 96)	PreviouslyDiagnosed(N = 252)	HIV Uninfected (N = 1167)	P value* (chisquare test)	P value**(chi square test)
**Age at enrollment**						
** 18–30 y.o.**	33%	33%	12%	38%	<0.001	0.386
**Median**	40	37	43	38		
**IQR**	27, 47	25, 44	38, 49	25, 47		
**Latino/Hispanic**	8%	8%	6%	8%	0.424	0.899
**US Born**	96%	99%	96%	95%	0.295†	0.119†
**Less than college** **education**	55%	65%	54%	54%	0.064	0.049
**Currently student**	20%	14%	19%	21%	0.227	0.076
**Currently working**	31%	21%	17%	35%	0.300	0.011
**Lack stable housing**	10%	6%	6%	11%	0.917	0.175
**Annual household income**					0.661	0.269
** <$9,999**	38%	42%	38%	38%		
** $10,000–49, 999**	50%	51%	56%	49%		
** >$50,000**	11%	7%	6%	13%		
**Insufficient Income**					0.482	0.030
** Never**	44%	52%	49%	43%		
** Once in awhile**	32%	34%	32%	32%		
** Fairly or very often**	24%	14%	19%	25%		
**Marital status**					0.978	0.732
** Married, have primary partner**	11%	10%	10%	12%		
** Single, divorced,** **widowed**	89%	89%	90%	88%		
**Enrollment city**					<0.001	<0.001
** New York**	20%	27%	19%	19%		
** Boston**	15%	6%	15%	16%		
** Washington**	14%	21%	13%	14%		
** Atlanta**	19%	26%	15%	19%		
** San Francisco**	13%	2%	9%	15%		
** Los Angeles**	19%	18%	30%	16%		

* Newly diagnosed with HIV compared to previously diagnosed.

** Newly diagnosed compared to HIV-uninfected.

† p-value from Fisher’s exact test.

### Sexual Behavior

Participants chose from a list of 12 sexual orientation identifiers, with multiple selections allowed. Thirty percent of all participants identified exclusively as gay or homosexual (no other orientation selected), 29% identified exclusively as bisexual and 3% identified as transgender (Table 2). Participants had a median of 5 sexual partners in the past 6 months, with 82% having two or more male partners. More than half (51%) reported unprotected receptive anal intercourse, and three-quarters reported unprotected insertive anal intercourse with a male partner; 44% reported having sex with a female partner, and 25% reported having sex with a transgender partner in the past six months. About thirty percent of the men indicated that they exchanged money, drugs, goods or shelter during the last anal sex episode. Although participants who were newly diagnosed with HIV when they entered the study had fewer partners that they knew to be HIV-infected than those who were previously diagnosed with HIV (*p = 0.0013*) or HIV-uninfected at study entry (*p = 0.0011*), they tended to have more HIV status unknown partners than the previously diagnosed (*p = 0.004*) or HIV-uninfected (*p<0.001*) participants. More than one-half (53.2%) of the previously diagnosed participants reported having unprotected sex with at least one HIV-infected partner in the prior 6 months compared to 37.9% of newly diagnosed participants and 14.2% of HIV-uninfected participants (*p<0.001*).

**Table 2:** Sexual identity and behavior of HPTN 061study participants by HIV status: Newly diagnosed versus previously diagnosed or HIV-uninfected.

Variable	TotalPrevalence	NewlyDiagnosed(N = 96)		PreviouslyDiagnosed(N = 252)	HIVUninfected(N = 1167)	P value*	P value**
**Sexual Orientation**						0.261	0.004
** Homosexual/Gay**	30%	42%		40%	26%		
** Exclusively Bisexual**	29%	26%		20%	31%		
** Other**	42%	32%		40%	43%		
**Identify as transgender**	3%	5%		4%	3%	0.776†	0.151†
**N partners in prior 6 months**						0.198	0.316
** Median**	5	5		4	5		
** IQR**	3, 9	3, 9		2, 7	3, 10		
**Two or more male** **partners in prior 6 months**	82%	85%		76%	83%	0.060	0.515
**Having any HIV+male partners**	22%	38%		53%	14%	0.011	<0.001
**N HIV- partners:**	1	1		0	1	0.0016^‡^	0.0256^‡^
**median/Range**	0–700	0–172		0–39	0–700		
**N HIV+ partners**	0	0		1	0	0.0013^‡^	<0.001^‡^
**Median/range**	0–100	0–30		0–54	0–100		
**N unknown status partners**	1	2		1	1		
**Median/Range**	0–202	0–91		0–55	0–202	0.0044^‡^	<0.001^‡^
**Unprotected receptive** **anal sex with male** **partner(s) in last 6 months**	51%	69%		61%	47%	0.144	<.001
**Unprotected insertive** **anal sex with male** **partner(s) in last 6 months**	75%	72%		69%	76%	0.605	0.435
**Female partner(s)** **in the last 6 months**	44%	32%	28%		49%	0.407	0.002
**Transgender partner(s)** **in the past 6 months**	25%	17%		13%	28%	0.319	0.018
**Receiving money, drugs** **goods or shelter** **during last anal sex**	22%	17%		20%	23%	0.550	0.173
**Providing money, drugs** **goods or shelter** **during last anal sex**	10%	10%		13%	10%	0.440	0.884

* Newly diagnosed with HIV compared to previously diagnosed.

** Newly diagnosed compared to HIV-uninfected.

† P-value from Fisher’s exact test.

Compared to the participants who were HIV-uninfected at study enrollment, participants who were newly diagnosed with HIV were more likely to identify exclusively as homosexual or gay (42% vs. 26%, *p* = 0.004), were less likely to identify as bisexual (26% vs. 31%, *p* = 0.004), were more likely to report unprotected receptive anal intercourse with a male partner in the prior six months (69% vs. 47%, *p*<0.001), and were less likely to report having female sex partners in the prior six months (32% vs. 49%, *p* = 0.002). They were also less likely to report having transgender partners within the prior six months (17% vs. 28%, *p* = 0.018).

### Sexually Transmitted Infections

Sixteen percent of HPTN 061 participants had at least one bacterial STI diagnosed at study enrollment (Table 3). Three percent of study participants were newly diagnosed with active syphilis, with another 4% having serological evidence of prior syphilis. The prevalence of urethral gonorrhea was 1%, rectal gonorrhea 3%, urethral chlamydia 2% and rectal chlamydia 6% among the men in the study. Compared to men who were previously diagnosed with HIV, men who were newly diagnosed with HIV were much more likely to have active syphilis (11% vs. 4%) and were less likely to have treated infections (5% vs. 12%, *p*<0.001). Newly diagnosed participants were more likely to have rectal gonorrhea (8% vs. 4%, *p* = 0.011) or rectal chlamydial infections (15% vs. 6%, *p* = 0.020) compared to men who were previously diagnosed with HIV. Compared to HIV-uninfected men, men who were newly diagnosed with HIV were much more likely to have new active syphilis infection (11% vs. 2%, *p*<0.001) as well as prior syphilis infection (5% vs. 3%, *p*<0.001) and were more likely to have rectal gonorrhea (8% vs. 3%, *p* = 0.004), rectal chlamydia (15% vs. 6%, *p*<0.001) and at least one sexual transmitted infection (31% vs. 14%, *p*<0.001).

**Table 3:** Sexually transmitted infections among Black men who have sex with men (participants in HPTN 061) by HIV status: Newly diagnosed versus previously diagnosed or HIV-uninfected at baseline (N = 1553).

Variable	TotalPrevalence	Newly HIV Diagnosed (N = 96)	Previously HIV Diagnosed (N = 252)	HIV Uninfected(N = 1167)	P value* (chisquare test)	P value**(chi square test)
**Syphilis**					<0.001	<0.001
**New active infection**	3%	11%	4%	2%		
**Treated infection**	4%	5%	12%	3%		
**Gonorrhea**						
**Urethral**	1%	1%	2%	1%	0.199	0.116
**Rectal**	3%	8%	4%	3%	0.011	0.004
**Chlamydia**						
**Urethral**	2%	4%	2%	2%	0.106	0.025
**Rectal**	6%	15%	6%	6%	0.020	<0.001
**Multiple STI**	3%	11%	3%	2%	0.010	<0.001
**Any STI**	16%	31%	25%	14%	0.209	<0.001

* Newly diagnosed with HIV compared to previously diagnosed.

** Newly diagnosed compared to HIV-uninfected.

### Psychosocial and Behavioral Issues

More than one-third of all participants (38%) reported using stimulants in the prior 6 months, and more than half (56%) reported using marijuana (Table 4). Participants also reported popper use (12%), opiate use (6%), and injecting drugs (5%). Almost half (47%) reported substance use during their last anal sex encounter. Forty-three percent of the men had medium to high levels of internalized homophobia, and 45% reported depressive symptoms. Forty-four percent reported that they were currently a member of a religious group and 76% said that they were a member of a religious institution while growing up (Table 4). Compared to men who were previously diagnosed with HIV, men who were newly diagnosed with HIV did not differ with regard to their prevalence of substance use, internalized homophobia, depression, or prior religious affiliation, but were less likely to be members of a religious institution growing up (70% vs. 81%, *p* = 0.030). Compared to individuals who were HIV-uninfected, men who were newly diagnosed with HIV did not differ with regard to substance use, internalized homophobia, depression, and current or prior religious affiliation.

**Table 4:** Social and behavioral issues among Black men who have sex with men (participants in HPTN 061) by HIV status: Newly diagnosed versus previously diagnosed or HIV-uninfected at baseline (N = 1553).

Variable	Total Prevalence	NewlyDiagnosed(N = 96)	Previously Diagnosed (N = 252)	HIV Uninfected (N = 1167)	P value* (chi square test)	P value** (chi square test)
**Drug use in last 6 months**						
**Injected Drugs**	5%	5%	4%	5%	0.206	0.187
**Any Opiates**	6%	3%	3%	6%	0.271	0.253
**Any Poppers**	12%	13%	20%	10%	0.134	0.384
**Any Stimulants**	38%	37%	41%	38%	0.573	0.878
**Any Marijuana**	56%	54%	53%	57%	0.861	0.642
**Substance use during last** **anal sex**	47%	39%	42%	48%	0.658	0.093
**Internalized homophobia**					0.575	0.356
** Low (7–16)**	57%	62%	68%	54%		
** Medium (17–26)**	35%	30%	27%	37%		
** High (27–35)**	8%	8%	5%	9%		
**Depressive symptoms**	45%	44%	49%	44%	0.472	0.929
**Member of a religious** **institution now**	44%	43%	54%	43%	0.083	0.921
**Member of a religious** **institution growing up**	76%	70%	81%	76%	0.030	0.212

* Newly diagnosed with HIV compared to previously diagnosed.

** Newly diagnosed compared to HIV-uninfected.

### Multivariate Logistic Regression Analyses

Compared to men who were previously diagnosed with HIV infection, men who were newly diagnosed with HIV infection were significantly more likely to be younger (adjusted OR [AOR] = 2.9, 95% CI: 1.5–5.6), and less likely to report HIV-infected partners (AOR = 0.5, 95% CI: 0.3–0.9)(Table 5). Compared to men who were HIV-uninfected at the time of enrollment, men who were newly diagnosed with HIV infection were significantly more likely to be unemployed (AOR = 2.6, 95% CI: 1.4–4.6), and less likely to have insufficient income often (AOR = 0.4, 95% CI: 0.2–0.9). Newly diagnosed participants were also more likely to have STIs compared to participants who were HIV-uninfected at the time of entry into the study (AOR = 2.3, 95% CI: 1.4–4.0). Newly diagnosed participants were also much more likely to engage in unprotected receptive anal intercourse with other male partners compared to HIV-uninfected men (AOR = 2.3, 95% CI: 1.4–3.8) and to have at least one HIV-infected partner (AOR = 3.8, 95% C.I. 2.3–6.3) (Table 6).

**Table 5:** Univariate and multivariate logistic regression on modeling the probability of Black men who have sex with men being newly diagnosed HIV infection (NHIV) versus previously diagnosed HIV infection (PHIV) at time of enrollment.

	Univariate Logistic Regression			Multivariate Logistic Regression*		
Characteristics	OddsRatio	95% CI	P-value	OddsRatio	95% CI	P-value
Age 18–30 vs. 30+	3.7	(2.1, 6.5)	<.001	2.9	(1.5, 5.6)	0.001
Education less than college vs. college or higher	1.6	(1.0, 2.6)	0.065	1.7	(1.0, 3.0)	0.057
Number of male partners 2+ vs. <2	1.8	(1.0, 3.5)	0.063	1.9	(0.9, 4.1)	0.084
Having HIV+male partners Any vs. none	0.5	(0.3, 0.9)	0.012	0.5	(0.3, 0.9)	0.026
Multiple STI Yes vs. no	4.0	(1.5, 10.1)	0.004	2.3	(0.8, 6.9)	0.146
Member of a church or religious/spiritual institutioncurrently No vs. yes	1.5	(0.9, 2.5)	0.084	1.2	(0.7, 2.0)	0.590
Member of religious/spiritual organization while growingup No vs. yes	1.8	(1.1, 3.1)	0.032	1.7	(0.9, 3.1)	0.118

* Multivariate regression model adjusted for city.

**Table 6:** Univariate and multivariate logistic regression on modeling the probability of Black men who have sex with men being newly diagnosed HIV infection (NHIV) versus HIV-uninfected at time of enrollment.

Prevalence	Univariate Logistic Regression			Multivariate Logistic Regression*		
Effect	OddsRatio	95% CI	ORP-value	OddsRatio	95% CI	ORP-value
Education Less than college vs. college or higher	1.5	(1.0, 2.4)	0.051	1.5	(0.9, 2.4)	0.125
Student status No vs. yes	1.7	(0.9, 3.1)	0.079	1.8	(0.9, 3.4)	0.076
Employment Not working vs. working	1.9	(1.1, 3.1)	0.012	2.6	(1.4, 4.6)	0.002
Insufficient Income Once in a while vs. never	0.9	(0.6, 1.4)	0.612	0.9	(0.5, 1.5)	0.710
Fairly often/very often vs. never	0.4	(0.2, 0.8)	0.010	0.4	(0.2, 0.9)	0.019
STI Any vs. none	2.9	(1.8, 4.6)	<.001	2.3	(1.4, 4.0)	0.002
Sexual Identity Selected Exclusively homosexual/gay vs. other	2.2	(1.3, 3.6)	0.002	1.4	(0.8, 2.5)	0.202
Exclusively bisexual vs. other	1.2	(0.7, 2.0)	0.560	1.6	(0.8, 3.0)	0.149
Having HIV+male partner Any vs. none	3.7	(2.3, 5.7)	<.001	3.8	(2.3, 6.3)	<.001
URAI Yes vs. no	2.6	(1.6, 4.1)	<.001	2.3	(1.4, 3.8)	0.002
Any female partner Yes vs. no	0.5	(0.3, 0.8)	0.002	0.7	(0.4, 1.4)	0.361
Any transgender partner Yes vs. no	0.5	(0.3, 0.9)	0.018	0.6	(0.3, 1.2)	0.151
Buzzed or drunk on alcohol with sex Yes vs. No	0.7	(0.5, 1.1)	0.094	0.8	(0.5, 1.4)	0.479

* Multivariate regression model adjusted for city.

## Discussion

In this analysis of one of the largest cohorts of Black MSM in the US, new HIV infection rates were high, and were associated with demographic (i.e., younger age), structural (e.g.lack of employment), biological (e.g., STIs), and behavioral (e.g., unprotected anal intercourse) factors compared with HIV-uninfected men. Prior research has indicated that Black MSM do not engage in greater levels of high-risk sex compared to other MSM [3]–[5], [10], but other factors may be potentiating HIV transmission, including lower levels of awareness of HIV status [11], delays in accessing clinical services [12], including antiretroviral treatment [6], [13]–[15], as well as higher rates of sexually transmitted infections [16], resulting in a relatively larger number of individuals who could transmit HIV, or who might be particularly susceptible to infection. Prior studies have suggested that Black MSM are more likely to select other Black MSM as sexual partners [5,6.17], thereby amplifying their risk with each unprotected sexual act [10]. Lower levels of serostatus awareness facilitate HIV spread since individuals who are HIV-infected and unaware of their serostatus are more likely to engage in unprotected sex than those who know they are infected [18]. In the current study, although Black MSM who were unaware of their infection had fewer known HIV-infected partners than HIV-uninfected participants, they tended to have more partners of unknown serostatus. This study enhances understanding of Black MSM’s disproportionate risk for HIV, by finding that those who were unemployed and those with untreated STIs were more likely to have undiagnosed HIV infection, raising the possibility that economically-related non-engagement with the health care system may enhance the impact of biological amplifiers of HIV transmission.

The majority of the men enrolled were economically disenfranchised, and this did not differ by infection status or serostatus knowledge: 38% had incomes of less than $10,000 per year (and 88% less than $50,000 per year), and 56% reported that their incomes were often or occasionally insufficient to meet household demands. Because income insecurity was so pervasive, it was not feasible to delineate its temporal association with HIV risk and transmission behavior. Economic insecurity has been associated with increased vulnerability to HIV in other studies [19.20], and may be associated with social immobility and lack of self-efficacy. Newly diagnosed men were more likely to be unemployed, which may result in delays in seeking health care services because of low income and lack of health insurance. It is noteworthy that homelessness was uncommon (only 10% of the sample), but this may be misleading, as the questionnaire did not ask about life time experience, nor did it ask about living with families, congregate living, or specifically about exchange of sex for housing. In the multivariate analysis, although men who were unemployed were more likely to have undiagnosed HIV infection, those who reported frequent insufficient income were less likely to have undiagnosed HIV infection. The reason for this seeming contradiction is unclear. Because both employed and unemployed study participants were poor, an independent relationship of income and risk could not be established. It is also possible that the poorest men in this sample had the least social mobility and opportunities to meet new partners. Further research is needed to understand the specific impacts that poverty and unemployment have in influencing sexual behavior in this population.

Newly diagnosed Black MSM tended to be younger, with one third being less than 30 years old, but were otherwise demographically similar to peers who had previously established HIV infection or who were uninfected. These data are consistent with surveillance data from the US Centers for Disease Control and Prevention (CDC) finding that Black youth have lower levels of serostatus awareness despite increased rates of new infections [1], highlighting the urgency of developing culturally-tailored prevention programs to increase testing among young Black MSM and their successful linkage to care [21], [22]. Less than one-third (30%) of Black MSM enrolled in HPTN 061 identified exclusively as gay or homosexual, and those who were newly diagnosed with HIV were more likely to choose those categories (42%) than Black MSM who were HIV-uninfected (26%), but most of the men (69%) who were infected reported engaging in unprotected receptive anal intercourse. Although newly diagnosed Black MSM were less likely to report having HIV-infected partners than those were previously diagnosed or who were HIV-uninfected, they reported having more unknown status partners than the other participants. These findings are consistent with other studies documenting high rates of HIV transmission in settings where HIV prevalence is high and a substantial number of men are unaware that they or their partners are HIV-infected [23], [24]. Prior work has found that Black MSM were less likely than White MSM engage in seroadaptive strategies to decrease their risk of HIV transmission or acquisition and more likely to report not knowing their partners’ serostatus, similar to the findings in this study [25], [26]. Culturally-tailored interventions designed to increase serostatus awareness, enhance condom use and provide chemoprophylaxis [26], [27] for high risk uninfected Black MSM, and earlier diagnosis and treatment for HIV-infected Black MSM [28], are needed to decrease HIV transmission in this population with a high community viral load. Newly diagnosed Black MSM were less likely to have female (32%) or transgender partners (17%) than those who remained HIV-uninfected (49% and 28%, respectively), but the level of contact with non-MSM partners underscores the need for prevention programs to focus on behaviors and not only on sexual identities [29].

Asymptomatic STIs were highly prevalent among the participants, with more than 16% having at least one bacterial STI at study entry. Black MSM who were unaware of their HIV infection were most likely to have undiagnosed STIs. The STI burden among participants in HPTN 061 may represent an underestimate since pharyngeal STI screening was not done in this study, but may not be reflective of STI prevalence among all Black MSM, since participants were selected based on their engaging in anal sex. Anorectal gonorrhea and chlamydia have been associated with increased risk for HIV acquisition [30]. In addition to potentiating HIV transmission and acquisition biologically due to mucosal inflammation [31], STIs are a marker for unprotected sex, selection of high-risk partners, and may also reflect lack of engagement in health care [12] due to economic vulnerability and/or medical mistrust [32]. The high rates of undiagnosed STIs and HIV infection seen in this study suggest that in order to improve the sexual health of Black MSM, careful assessments of sexual risk and comprehensive screening for STIs should become routine. Although substance use was commonly reported in HPTN 061, no specific drugs were associated with being newly or previously diagnosed with HIV infection, but large subsets of infected and uninfected Black MSM reported using drugs in this study, with more than half reporting marijuana use and more than one third stimulant use. Prior studies have suggested that drug treatment programs that are tailored for MSM may decrease HIV risk taking among stimulant using MSM [33], [34]; further cultural adaptation for Black MSM may enhance the uptake and acceptance of these interventions.

Black MSM have reported multiple manifestations of stigma and discrimination (e.g., homophobia and racism) and that these socio-cultural factors may influence their mental health and HIV risk behaviors [15], [35]. Although levels of depressive symptoms and internalized homophobia did not differ between HIV-infected and uninfected Black MSM, they were very common, with 45% of the men in HPTN 061 reporting depressive symptomatology, and 43% noting increased levels of internalized homophobia. Interventions focused on reducing the disproportionate burden of HIV among Black MSM should screen for depression and provide psychosocial support to mitigate stressors related to stigma and discrimination [16], [36], [37]. Internalized homophobia may be a result of growing up in non-affirming communities, and has been linked to increased sexual risk taking behaviors [38], often in conjunction with depression and other affective disorders [39]. Interventions that build on the resilience that many MSM develop in the face of lack of social support in their home communities may facilitate HIV prevention efforts by enhancing self-efficacy [40], [41]. The majority of participants reported a religious identification when growing up (76%) and 44% of the whole cohort indicated they were currently involved in a faith community. Prevention interventions for Black MSM that engage faith leaders and religious communities may be helpful in decreasing self-stigmatization and could enhance willingness to engage in care [41], [42].

Although the study population was large and geographically diverse, the study had its limitations, due to how the sample was derived and the protocol’s design. Some of the differences in HIV prevalence between cities may be a result of recruitment efforts that included diverse approaches, and were not designed to weight subsets of Black MSM to draw wider community inferences. The enrollment of participants who were HIV-uninfected was capped at 200 participants at each site, and an enrollment cap of 10 was applied to community-recruited participants with a prior diagnosis of HIV infection who were already in care, or reported only having unprotected anal sex with HIV-infected partners, since they would be at decreased risk for HIV transmission. Individuals could refer no more than five partners into the study, and over the course of the study, the average number of referrals was less than 1 eligible participant. The limited referral rates may reflect residual stigma regarding HIV and acknowledging same sex behavior. The cross-sectional nature of this analysis limits inferences regarding temporal relationships. For all these reasons, the data must be interpreted carefully, without making broad generalizations about all Black MSM. Nonetheless, the sample size of 1553 participants recruited in 6 cities in very different parts of the country represents one of the largest prospective studies of Black MSM, and the extensive data represent a resource to enhance understanding of the factors associated with the disproportionate HIV epidemic in this population.

In summary, these data from HPTN 061 build on prior formative research focused on the significant and widespread HIV epidemic among Black MSM in the US. Since unprotected receptive anal intercourse was the major mode of HIV transmission among the men, studies of how to best promote sexual health for Black MSM are needed. Further research designed to develop prevention interventions for Black MSM should also assess whether addressing socioeconomic, behavioral and broader health care concerns may lead to decreases in HIV incidence in this heavily impacted population.

## Supporting Information

File S1 Appendix.
(DOCX)
