# Supplementary material for: Concomitant Socioeconomic, Behavioral, and Biological Factors Associated with the Disproportionate HIV Infection Burden among Black Men Who Have Sex with Men in 6 U.S. Cities
Source: PLoS One. 2014 Jan 31;9(1):e87298. doi: 10.1371/journal.pone.0087298 (PMC3909083; doi:10.1371/journal.pone.0087298)
Supplement: File S1 — Appendix. (DOCX) [file pone.0087298.s001.docx]

File S1 - Appendix

Between July 2009 and October 2010, Black MSM were enrolled in 6 US cities to evaluate the feasibility of a multi-component prevention intervention. This analysis focuses on the correlates of being newly diagnosed with HIV compared to being HIV uninfected or previously diagnosed with HIV, using multivariate logistic regression to delineate key factors.

The institutional review boards at all participating institutions approved the study.

Harlem Prevention Center CRS - Columbia University Medical Center Institutional Review Board; Located at: New York, New York

NY Blood Ctr./Union Square CRS - IRB00000068 - New York Blood Ctr IRB #1; Located at: New York, NY

George Washington University CRS - IRB00000169 - George Washington U Med Ctr IRB #1; Located at: Washington, DC

San Francisco Vaccine and Prevention CRS - IRB00000230 - San Francisco General Hosp (SFGH) Committee IRB #2; Located at: 333 California Street, Suite 315, University of California San Francisco, CA 94118

The Ponce de Leon Ctr. CRS - IRB00000569 - Emory U IRB #2 – Biomedical IRB (Cmte A); Located at: Atlanta, GA AND Grady Research Over Site Committee (GROC) Hope Clinic of the Emory Vaccine Center CRS - IRB00000569 - Emory U IRB #2 - Biomedical IRB (Cmte A); Located at: Atlanta, GEORGIA

UCLA Vine Street CRS -IRB# 00004474 - University of California, Los Angeles - South General Campus IRB (SGIRB); Located at: Los Angeles, CA

Fenway Institute CRS - IRB00000858 – Fenway Community Health IRB #1; Located at: Boston, Massachusetts.

At the enrollment visit, eligibility was confirmed and written informed consent obtained.

This list of IRBs is also included as an appendix.
